# Functional analysis of the omega-6 fatty acid desaturase (*CaFAD2*) gene family of the oil seed crop *Crambe abyssinica*

**DOI:** 10.1186/1471-2229-13-146

**Published:** 2013-10-01

**Authors:** Jihua Cheng, Li-Hua Zhu, Elma MJ Salentijn, Bangquan Huang, Jens Gruber, Annemarie C Dechesne, Frans A Krens, Weicong Qi, Richard GF Visser, Eibertus N van Loo

**Affiliations:** 1 Wageningen UR Plant Breeding, P.O. Box 16, 6700, AA Wageningen, The Netherlands; 2 College of Life Science, Hubei University, Wuhan, People’s Republic of China; 3 Plant Breeding and Biotechnology, Swedish University of Agricultural Science, Alnarp, Sweden; 4 Institute for Biology I-Botany, RWTH Aachen University, Aachen, Germany

**Keywords:** Crambe abyssinica, Fatty acid desaturase 2, Oil crop, Oleic acid, Polyunsaturated fatty acid, RNAi, Gene expression

## Abstract

**Background:**

*Crambe abyssinica* produces high erucic acid (C22:1, 55-60%) in the seed oil, which can be further increased by reduction of polyunsaturated fatty acid (PUFA) levels. The omega-6 fatty acid desaturase enzyme (FAD2) is known to be involved in PUFA biosynthesis. In crambe, three *CaFAD2* genes, *CaFAD2-C1, CaFAD2-C2* and *CaFAD2-C3* are expressed.

**Results:**

The individual effect of each *CaFAD2* gene on oil composition was investigated through studying transgenic lines (*CaFAD2-RNAi*) for differential expression levels in relation to the composition of seed-oil. Six first generation transgenic plants (T_1_) showed C18:1 increase (by 6% to 10.5%) and PUFA reduction (by 8.6% to 10.2%). The silencing effect in these T_1_-plants ranged from the moderate silencing (40% to 50% reduction) of all three *CaFAD2* genes to strong silencing (95% reduction) of *CaFAD2-C3* alone. The progeny of two T_1_-plants (WG4-4 and WG19-6) was further analysed. Four or five transgene insertions are characterized in the progeny (T_2_) of WG19-6 in contrast to a single insertion in the T_2_ progeny of WG4-4. For the individual T_2_-plants of both families (WG19-6 and WG4-4), seed-specific silencing of *CaFAD2-C1* and *CaFAD2-C2* was observed in several individual T_2_-plants but, on average in both families, the level of silencing of these genes was not significant. A significant reduction in expression level (*P* < 0.01) in both families was only observed for *CaFAD2-C3* together with significantly different C18:1 and PUFA levels in oil.

**Conclusions:**

*CaFAD2-C3* expression is highly correlated to levels of C18:1 (r = -0.78) and PUFA (r = 0.75), which suggests that *CaFAD2-C3* is the most important one for changing the oil composition of crambe.

## Background

Crambe (*Crambe abyssinica* Hochst ex. R. E. Fr.) belongs to the *Brassicaceae* plant family. The seed oil of crambe contains a high content of erucic acid (C22:1, 55% to 60%) and this oil thus has applications as industrial oil [1]. Erucic acid in form of erucamide, a major derivative from C22:1, can be used as slip agent in plastics, or lubricants, nylon and cosmetics [2]. More recently, crambe oil is considered to be potential to produce biofuel [3]. The cultivation of crambe yields up to 1 t ha^-1^ of oil, comparative to that of high erucic acid rapeseed (HEAR). Furthermore, the processing costs for crambe oil extraction are in the same range of that for rapeseed [1,4,5]. The high yield and the fact that crambe is not able to cross with food oil crops in nature make crambe an ideal platform to produce industrial oils [6,7]. In addition to high erucic acid (C22:1), crambe oil contains also certain amounts of oleic acid (C18:1), linoleic acid (C18:2) and linolenic acid (C18:3).

The value of crambe oil can be further improved not only by increasing the C22:1 content but also by reducing the content of polyunsaturated fatty acids (PUFA, C18:2 + C18:3). Firstly, an increase in C22:1 can reduce the purification cost of C22:1 from C18-fatty acids. It was estimated that 10% increase of C22:1 in oil would reduce the processing costs by half [8]. Secondly, reduction in PUFA is beneficial for storage and extending the shelf life of the oil because PUFA are highly prone to oxidation during storage[9]. Thirdly, high PUFA content causes higher viscosity for the oil (a disadvantage to biodiesel) [10]. Considerable efforts have been made to reduce PUFA in oil. A common chemical approach is to reduce the PUFA content or increase the C18:1 content through hydrogenation [9,11]. However, this hydrogenating process is expensive and adds extra 2 to 3 cent per pound cost to the price of oil [12].

Molecular breeding approaches to change composition of seed oil are targeting important genes involved in the fatty acid biosynthesis pathways, which have been intensively studied and many genes involved have been characterized. In short, C18:1 is *de novo* synthesized in plastids and transported into the endoplasmic reticulum where the C18:1 is incorporated into phosphatidylcholine (PC) and may undergo desaturation to C18:2 and next to C18:3 by the actions of two microsomal enzymes; delta-12-fatty acid desaturase (FAD2) [omega-6 desaturase] and delta-15-fatty acid desaturase (FAD3) [omega-3 desaturase], respectively [9,13]. Alternatively, C18:1 may undergo elongation to very long chain fatty acids (VLCFA, C22:1 for example) by the action of fatty acid elongation (FAE) complex [2,14-17]. Mutant and genetic mapping studies showed that the enzyme FAD2 was found to be mainly responsible for C18:1 and PUFA content although FAD3 also contributes to a limited degree to these traits [9,18]. In addition, the acyl flux between the two pathways, the prokaryotic (plastidial) and eukaryotic (mainly in the ER) pathway, is influencing the C18:1 pool [19-21].

Genetic modification (GM) aimed at regulating the *FAD2* expression has been applied to produce oils with higher C18:1 in various oil crops [8,22,23]. For example, by anti-sense suppression of *FAD2* in *Brassica juncea*, a transgenic line was obtained that produced oil with higher C18:1 (73%) and lower PUFA (8% of C18:2; 9% of C18:3) compared to the wild type (53% of C18:1; 24% of C18:2; 16% of C18:3) [23]. Similarly, a significant change in C18:1 and PUFA and even a C22:1 increase in the seed oil was observed when silencing *FAD2* with both co-suppression and anti-sense in *Brassica carinata*[8]. Gene silencing by RNAi has been considered to be a particularly efficient way to obtain stable transgenic plants with the silenced target genes [24,25]. RNAi-mediated silencing of *GhFAD2* in cotton enabled over 60% increase in C18:1 [22]. Simultaneous RNAi-mediated silencing of *FAD2* and *FAE1* in *Brassica napus* caused not only significant increase in C18:1 (from 62% to 85%), but also reduction in C22:1 and PUFA (from 26% to 10% and from 0.87% to 0% respectively) [26]. Recently, several efficient protocols for crambe transformation are available [27,28] and RNAi has shown to be an effective gene knockdown tool for crambe where *CaFAD2* RNAi gene silencing resulted in increased C18:1 levels (from 14.5% to 24.9%) [29]. Introduction of two heterologous genes, *LdLPAAT* and *BnFAE1*, in such *CaFAD2*-RNAi lines directed the oil biosynthesis towards the incorporation of C22:1 at the *sn*-2 position of triacylglycerol, thereby the C22:1 level increased from 60% in the wild type to 73% in the best transgenic crambe line [29].

An obstacle to adaptation of such genetic modified (GM) crops is the lack of broad acceptance by a part of the community in many countries [30]. Furthermore, there are some cases where RNAi-mediated traits are not completely reliable on the long term in generating stable target gene suppression [25]. Currently, breeding of the allo-hexaploid crambe mainly relies on traditional approaches, however, the possibilities are restricted by the lack of genetic variation for important agronomic traits [1,31,32]. In such situations and particularly when genes controlling a phenotype are known, mutation breeding of induced or natural mutations, identified via “TILLING” (Targeting Induced Local Lesions IN Genomes) [33], offers a reliable, stable, non-GM approach to obtain the desired oil quality in crambe. A drawback is that “TILLING” is still a challenging task in polyploid crops, where the multiple alleles are creating problems in identifying desirable genetic changes due to gene redundancy. Despite these problems successful cases of targeted mutagenesis have been reported in polyploid crops [34-38]. For instance, targeted mutation breeding of natural or induced variation in the *FAD2* gene or mining natural variants has been used to develop crops with high C22:1 or less PUFA [38-40]. By combining mutations or natural variants of *FAD2* and *FAD3*, it was possible to produce oils with higher C18:1 and lower C18:3 in soybean and *Brassica napus*[9,11]. In the allo-hexaploid genome of *Crambe abyssinica* cv. 'Galactica’ seven *FAD2* genes are present, of which only three are transcriptionally active throughout plant development (*CaFAD2-C1, CaFAD2-C2* and *CaFAD2-C3*) [32]. The possibility of functional redundancy among the active *CaFAD2* family members may complicate the gain of crambe lines with desirable oil composition via mutation breeding.

The aim of the present study is to specify which of the *FAD2* genes in crambe is the key gene for increasing the C18:1 level, but reducing the PUFA content. *FAD2*-RNAi lines of crambe cv. 'Galactica’ were studied for functional correlations between the individual *CaFAD2* family members, *CaFAD2-C1*, *CaFAD2-C2* and *CaFAD2-C3* and seed-oil composition. Seed-oil composition and gene expression studies were performed in two independent families of the second generation transgenic lines (T_2_-plants). In addition, two other genes, *CaFAD3* and *CaFAE1* that are acting in close connection to *CaFAD2*, were also involved in the study.

## Results

### CaFAD2-RNAi lines

We have previously shown that the major expressed *CaFAD2* gene in developing crambe seeds is *CaFAD2-C3,* while the other two genes *CaFAD2-C1* and *CaFAD2-C2* are naturally expressed to much lower levels (4 and 100 times lower than *CaFAD2-C3* respectively) [32]. A DNA fragment of *CaFAD2-C2*, sharing 97% and 96% nucleotide identity with *CaFAD2-C1* and *CaFAD2-C3* respectively, was used to trigger RNAi-mediated silencing of the *CaFAD2* genes in crambe. Two primary transgenic plants (T_0_ generation,WG4 and WG19) that showed significantly lower contents of C18:2 and C18:3, but higher level of C18:1 in the seed-oil were used in this study [29]. For these two independent transgenic lines, the effect of RNAi-silencing on the expression of the individual *CaFAD2* genes and the composition of the seed-oil were analysed for the seeds produced by the first generation (T_1_) and the second generation (T_2_). In the T_2_ generation the transgene copy number was determined and the relative gene-expression of the individual genes was correlated to differences in seed-oil composition to determine the effect of silencing of the individual *CaFAD2* gene(s).

### *CaFAD2* gene-silencing and oil composition in T_1_-generation

**Figure 1:**
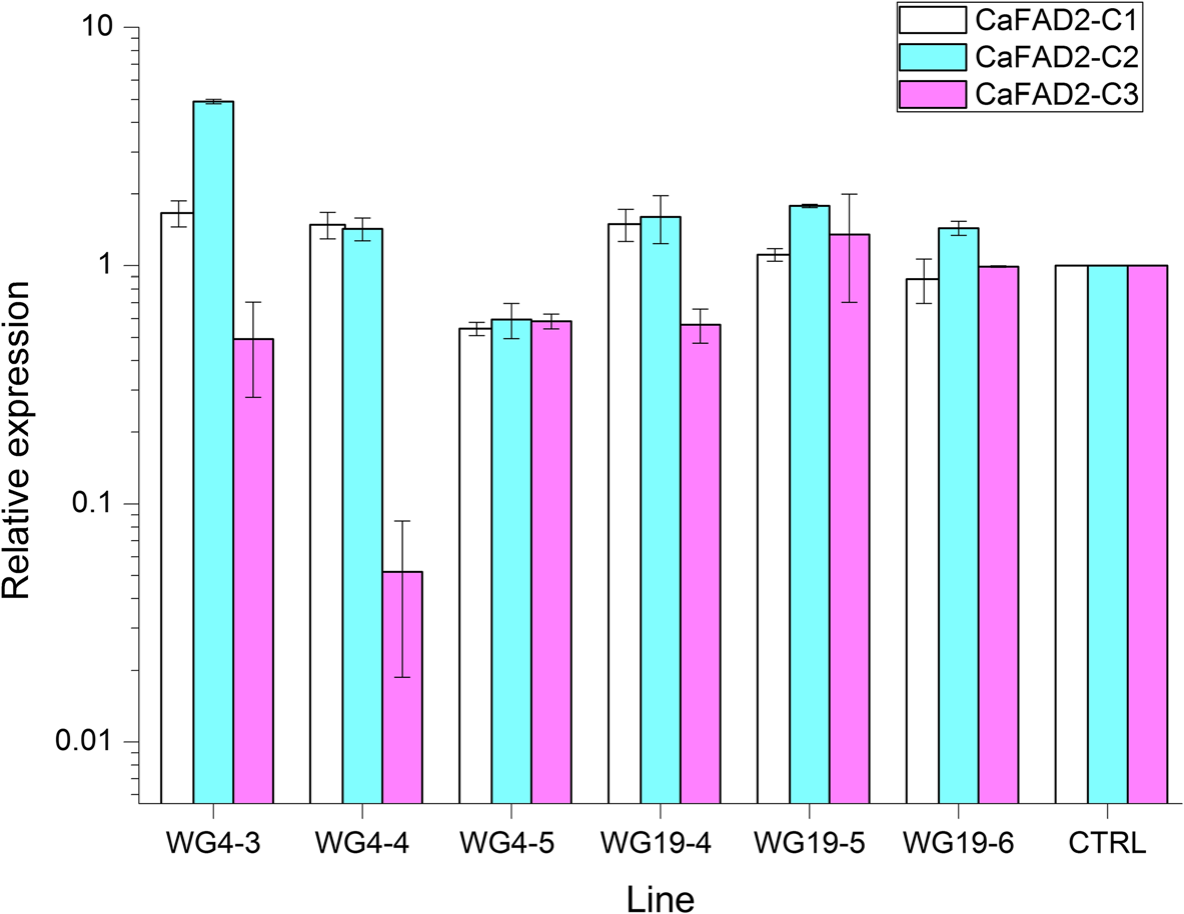
**Relative expression levels of *CaFAD2 *gene family members in the seeds of T**_**1 **_**plants of *C. abyssinica*.** Six T_1_ plants (WG4-3, WG4-4, WG4-5, WG19-4, WG19-5 and WG19-6) and a control (CTRL) were measured. Qantitification was performed by qPCR for bulk seeds (five to ten, 20 DAP) of each plant. The relative expression levels in the seeds of T_1_ plants were calculated with 2^-∆∆Ct method using β-*ACT2* as a reference gene.

Among six individual plants analysed, simultaneous but moderate silencing of all three *CaFAD2* genes (40% to 50% reduction) was found only in one plant, WG4-5. In two plants WG19-5 and WG19-6 no signs of gene-silencing was observed. In the remaining three plants (WG4-3, WG4-4 and WG19-4) no silencing was detected for *CaFAD2-C1* and *CaFAD2-C2* whereas *CaFAD2-C3* was silenced to different levels whereby a strong silencing of *CaFAD2-C3* (95% reduction) was detected in plant WG4-4 followed by a moderate level of *CaFAD2-C3* silencing (40% to 50% reduction) in WG4-3 and WG19-4 (Figure 1). To measure the final effect of gene-silencing, the oil composition was determined for around 20 single ripe seeds per plant. Despite the fact that clear silencing was only detected in the developing seeds of one plant (WG4-4), the contents of C18:1, C18:2 and C18:3 in the seeds of all six plants were significantly different to that of the control (Table 1). The C18:1 content was found to be 6% to 10.5% above the control, and the content of PUFA (C18:2 + C18:3) was a concomitant 4.3% to 9.2% lower than the control. Regarding C22:1 content, the plants WG4-3, WG4-4 and WG19-6 scored significantly higher than the control by respectively 2.4% (*P* < 0.01), 3.9% (*P* < 0.01) and 3.1% (*P* < 0.01) whereas the remaining three plants showed no significant difference to the control (Table 1).

**Table 1:** **Oil composition (%) in seeds of T**_**1 **_**lines of *C. abyssinica***

**ID**	**No. of seed**	**FA level**	**Oleic acid (C18:1)**	**Linoleic acid (C18:2)**	**Linolenic acid (C18:3)**	**Erucic acid (C22:1 )**
**WG4-3**	18	Mean ± SD ^a^	20.1 ± 2.4**	2.7 ± 0.9**	3.0 ± 0.8**	65.5 ± 3.4**
		Max	26.3	4.7	5.4	69.3
		Min	16.8	1.6	1.9	54.6
**WG4-4**	20	Mean ± SD	20.0 ± 1.1**	2.3 ± 0.5**	3.0 ± 0.5**	67 ± 2.5**
		Max	21.9	3.3	4.1	70.8
		Min	18.0	1.5	2.3	60.7
**WG4-5**	19	Mean ± SD	21.9 ± 3.5**	2.1 ± 1.8**	2.3 ± 0.9**	63.5 ± 3.2
		Max	27.4	7.6	5.4	66.6
		Min	11.5	1.0	1.5	55.5
**WG19-4**	19	Mean ± SD	22.6 ± 2.3**	1.6 ± 0.2**	2.5 ± 0.3**	60.7 ± 5.2
		Max	29.5	2.1	3.1	65.9
		Min	20.4	1.3	1.8	46.1
**WG19-5**	20	Mean ± SD	21.4 ± 3.2**	2.7 ± 1.8**	3.3 ± 0.7*	60 ± 4.6
		Max	27.1	9.5	5.3	64.1
		Min	11.3	1.3	2.3	50.2
**WG19-6**	20	Mean ± SD	18.0 ± 0.9*	2.0 ± 0.4**	2.4 ± 0.3**	66.2 ± 1.0**
		Max	20.0	2.9	3.0	68.0
		Min	16.4	1.4	1.9	64.5
**Control**	19	Mean ± SD	12.0 ± 0.5	8.2 ± 0.4	5.1 ± 0.2	63.1 ± 0.8
		Max	12.8	9.0	5.6	64.4
		Min	10.9	7.5	4.7	61.6

The fatty acid composition in single seed was measured, and for each line several seeds were used for measurement. Kruskal-Wallis test was run to determine significance level of difference between the FAD2-RNAi lines and the control. a: SD = standard deviation. Significance levels: *P < 0.05 and **P < 0.01.

An explanation for the fact that the level of *CaFAD2* silencing and the oil composition are not clearly correlated in T_1_ generation may reside in differences in zygosity level of the transgene copies among the T_1_-plants. As the expression profile was analysed on bulks of five to ten seeds, variation in transgene copy number and the presence of seeds that have segregated to the wild-type may mask the silencing effect. Indeed, among the fatty acid profiles of the individual seeds of WG4-5 and WG19-5 segregation to the wild-type oil composition was observed (Additional file 1).

### *CaFAD2* silencing and oil composition in T_2_-generation

Two T_1_-plants (WG4-4 and WG19-6) with stable and high C18:1 content were chosen to develop a second generation for an extensive study of the inheritance of the “high C18:1, low PUFA” oil-phenotype and to examine the correlation between silencing of the respective *CaFAD2* genes, the oil composition and the expression of two other genes involved in the seed oil biosynthesis (*CaFAD3* and *CaFAE1*).

#### Transgene copy-number

Seventeen T_2_-plants (5 from WG4-4 and 12 from WG19-6, designated as family WG4-4 and WG19-6) were randomly selected and characterised for the number of transgene insertion by Southern analysis. Four or five transgene insertions were detected in the progeny of WG19-6 with a similar pattern. In contrast, a single insertion was detected in all five analysed progeny plants of WG4-4 (Additional file 2).

#### CaFAD2 gene-silencing

**Figure 2:**
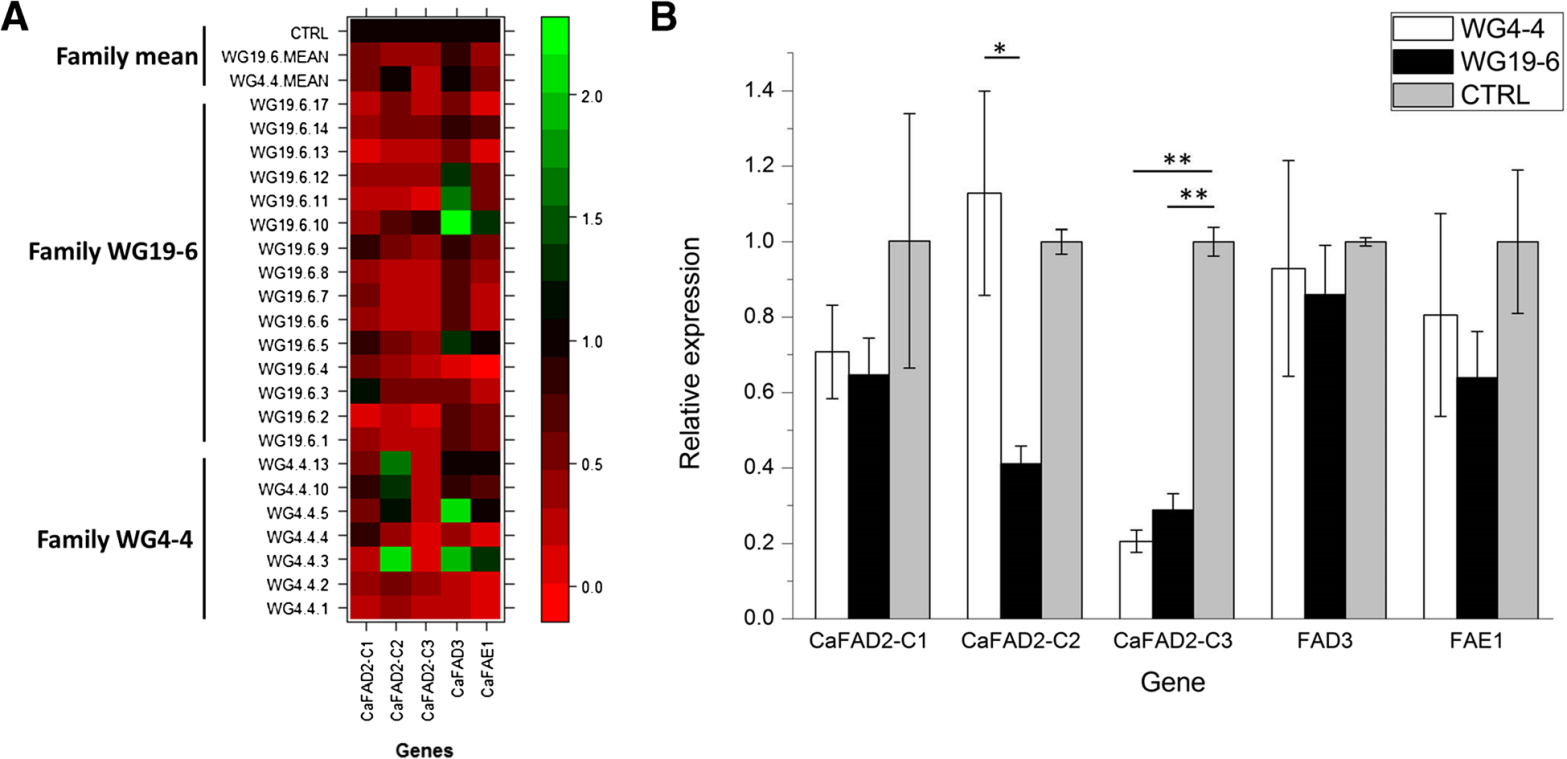
**Relative expression levels of *CaFAD2 *gene family members, *CaFAD3 *and *CaFAE1 *in the seeds of T**_**2 **_**plants of *C. abyssinica*. A)** Heatmap of the gene expression profiles in the seeds of T_2_ plants. The data indicate relative expression value of five genes (*CaFAD2-C1*, *CaFAD2-C2*, *CaFAD2-C3*, *FAD3* and *FAE1*, in horizontal) in bulk seeds (five to ten) at 20 DAP compared to the control (CTRL). Measurements were carried out for 21 progenies of two families (WG4-4 and WG19-6) and two control plants. The colours represent up-regulation or down-regulation (green, up; red, down). The colour key represents the scale of expression regulation. **B)** Comparison of the relative expression levels among the two families and the control. The data indicate the averages of gene expression levels in the seeds of T_2_ plants. The comparison was run with non-parametric test Kruskal-Wallis. Only the significant differences were shown. *, *P* < 0.05; **, *P* < 0.01.

To test whether and to what extent RNAi-mediated gene-silencing was effective in the T_2_-plants, the expression levels of *CaFAD2-C1*, *CaFAD2-C2* and *CaFAD2-C3* in developing seeds (five to ten bulked seeds per plant, 20 DAP) were quantified for all twenty-two T_2_-plants by qRT-PCR. The relative expression levels were calculated relative to the average expression level of the respective genes in the control. Collectively, down-regulation of gene expression is clearly detectable in the T_2_-plants of both T_2_ families, WG4-4 and WG19-6, differential patterns of silencing are observed for all three *CaFAD2* genes (Figure 2A). The major *CaFAD2* gene expressed in crambe seed, *CaFAD2-C3,* is strongly silenced (> 50% reduction) in all seven T_2_-plants of the WG4-4 family and in 14 out of 15 T_2_-plants of the WG19-6 family. Only in a single plant, WG19-6-10, minor silencing of *CaFAD2-C3* is observed (~10% reduction) (Figure 2A).

In contrast to the constant and strong silencing of *CaFAD2-C3*, more variation in silencing is found for *CaFAD2-C2* and *CaFAD2-C1* in both families. In the single copy transgenic T_2_-plants of the WG4-4 family, *CaFAD2-C2* showed strong silencing in almost half (3 out of 7) of the WG4-4 family (WG4-4-1, WG4-4-2 and WG4-4-4) whereas in the other T_2_-plants of WG4-4 this gene was not silenced (Figure 2A). In the other family, WG19-6, 60% of the T_2_-plants (9 out of 15) showed strong silencing of *CaFAD2-C2* whereas the rest showed zero- to moderate silencing of *CaFAD2-C2.* Also, *CaFAD2-C1* showed various silencing levels in both T_2_ families with strong silencing in three WG4-4 T_2_ -plants (3 out of 7; 43%) and five WG19-6 T_2_-plants (5 out of 15; 33%) (Figure 2A).

The average expression levels of the respective genes were calculated for each T_2_ family in comparison with the control. In both families (WG4-4 and WG19-6), only the expression of *CaFAD2-C3* is constantly and significantly different to the average control level (Figure 2B). For *CaFAD2-C1* and *CaFAD2-C2,* the average expression levels in both families are not significantly different from the average expression level observed in the control (Figure 2B).

#### Inheritance of CaFAD2 silencing

As compared over two generations (T_1_ and T_2_), the level of silencing in WG19-6, the line which carries multiple transgene insertions, increased from zero silencing in the T_1_ to ~40%, 60% and 70% reduction for respectively *CaFAD2-C1*, *-C2* and *-C3* in the T_2_. Over two generations, T_1_ and T_2,_ of the single copy transgene WG4-4 the expression patterns of the *CaFAD2* genes were similar. In both generations, only *CaFAD2-C3* was dominantly down-regulated in expression (Figure 1 and 2B).

#### Effect of CaFAD2 silencing on CaFAD3 and CaFAE1 expression

As a consequence of *CaFAD2* gene silencing, and accompanying changes in the substrate flow in the oil biosynthesis pathway, the expression of other genes acting in the pathway may change. Therefore, the expression of two genes, *FAD3,* involved in the conversion of C18:2 to C18:3, and *FAE1*, acting in the production of very long chain fatty acids (i.e. C22:1) by chain-elongation of C18:1, were studied in the two T_2_ families (WG4-4 and WG19-6) and the control. Both genes showed differential expression among the individual plants within families, but on average no significant differences were observed between the two T_2_ families and the control (Figure 2B). Notably, *CaFAD3* and *CaFAE1* showed a similar regulation pattern because both of them were up- or down-regulated within the same plant with exception of two plants, WG19-6-11 and WG19-6-12 (Figure 2A).

#### Oil composition

**Figure 3:**
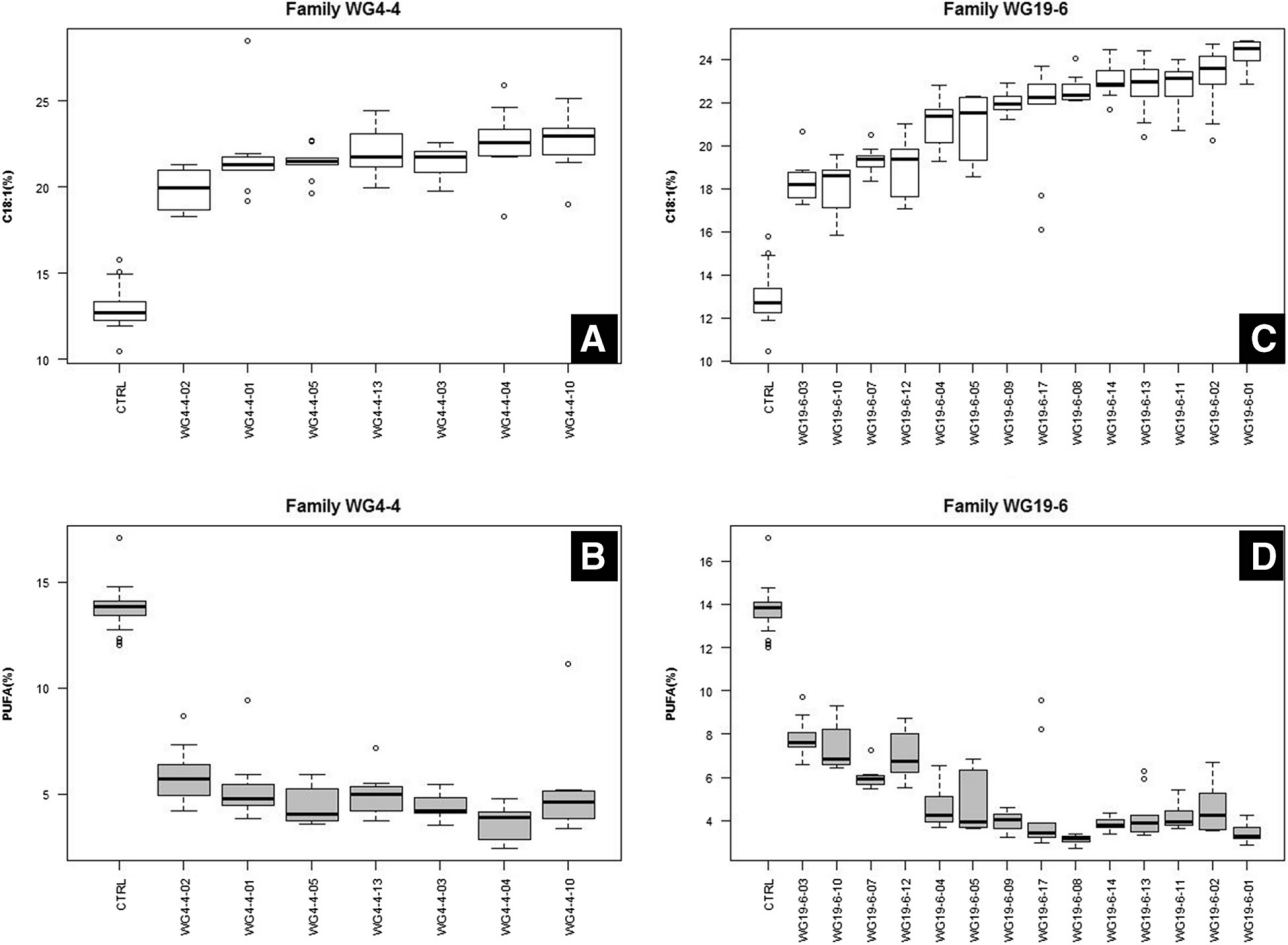
**Boxplots of C18:1 (A and C) and PUFA (B and D) levels in the seeds of T**_**2 **_**plants of *C. abyssinica*.** Each box indicates a T_2_ plant; ten single seeds per plant were measured.

The observed changes in expression pattern of the target genes resulted in significant changes in seed oil composition in 21 T_2_-plants studied (7 plants derived from WG4-4 and 14 from WG19-6). In accordance with the results found in the T_1_ generation, the contents of C18:1, C18:2 and C18:3 showed significant differences to the control (*P* < 0.01). However, the high C22:1 content found in the oil of two T_1_-plants (WG4-4 and WG19-6) was not observed throughout the entire T_2_ offspring of these plants. The C22:1 content showed significant differences to the control in only four T_2_-plants (WG19-6-3, WG19-6-5, WG19-6-7, WG19-6-8) in which C22:1 levels were about 2% higher (*P* < 0.05) than the control level (C22:1% = 62.5%). Compared to the control (C18:1% = 12.9%), the C18:1 contents in the oil of all the T_2_-plants were clearly higher (*P* < 0.01) and ranged from 24.3% to 18.2%, and most of the T_2_-plants (81%, 17 out of 21) showed a C18:1 content of more than 20% (Figure 3A and C). Consequently, the PUFA (C18:2 + C18:3) contents in these plants were lower (*P* < 0.01) than the control (PUFA% = 13.8%) and ranged from 3.4% to 7.9%, and in 67% (14 out of 21) of the plants the PUFA content was below 5% (Figure 3B and D).

**Figure 4:**
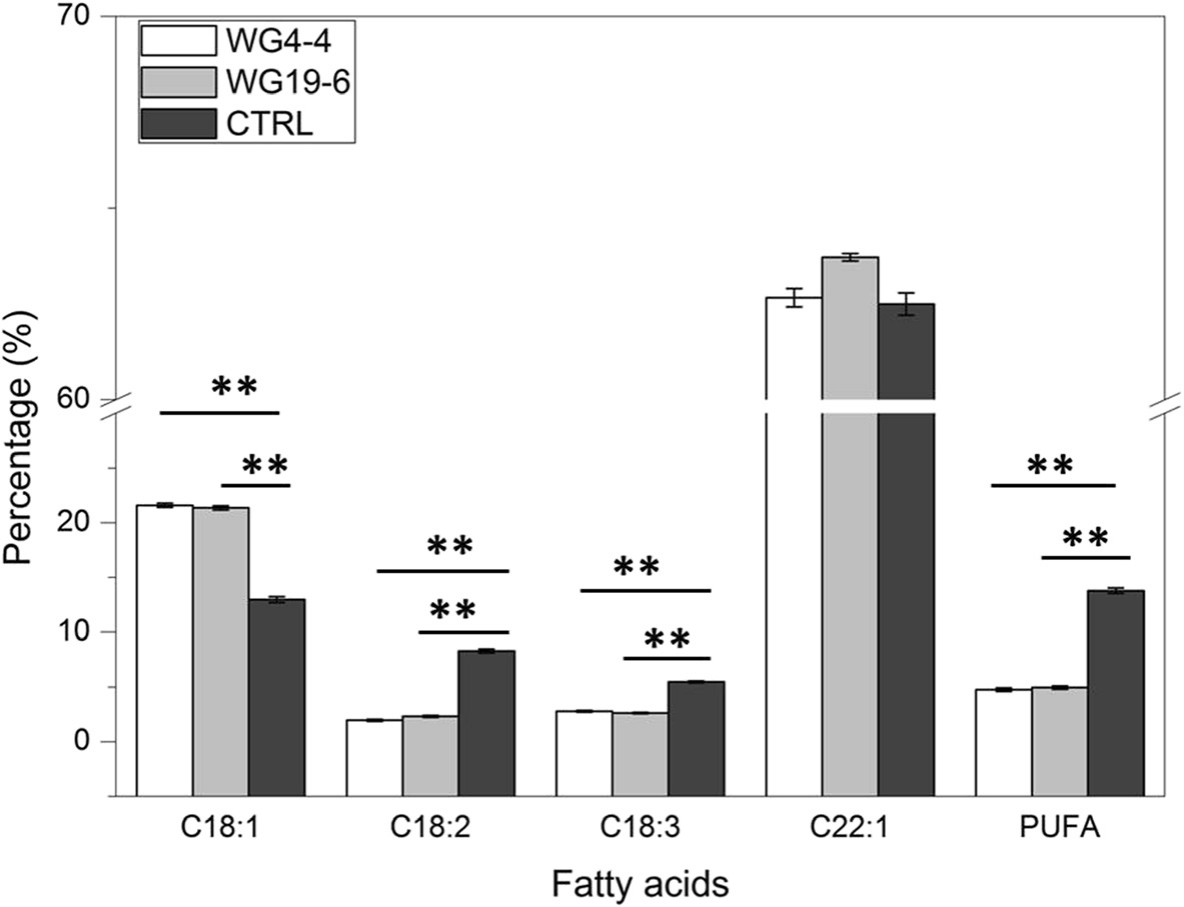
**Comparison of the oil composition in the seeds of T**_**2 **_**family WG4-4, WG19-6 and control plants of *C. abyssinica*.** The data represent the average of the two families and the control. For each plant, ten single seeds were measured. The bars indicate the comparisons with significant difference. **, *P* < 0.01. Error bar = standard error of mean.

Overall, seeds from the two T_2_ families (WG4-4 and WG19-6) contained different C18:1 and PUFA contents compared to the control (higher or lower respectively) (Figure 4). Among both T_2_ families, the content of C18:1, C18:2, C18:3, C22:1 and PUFA showed no significant difference (Figure 4). However, family WG19-6 showed more variation in both C18:1 and PUFA contents than family WG4-4 (Additional file 3).

#### Correlation between gene expression and oil composition

**Figure 5:**
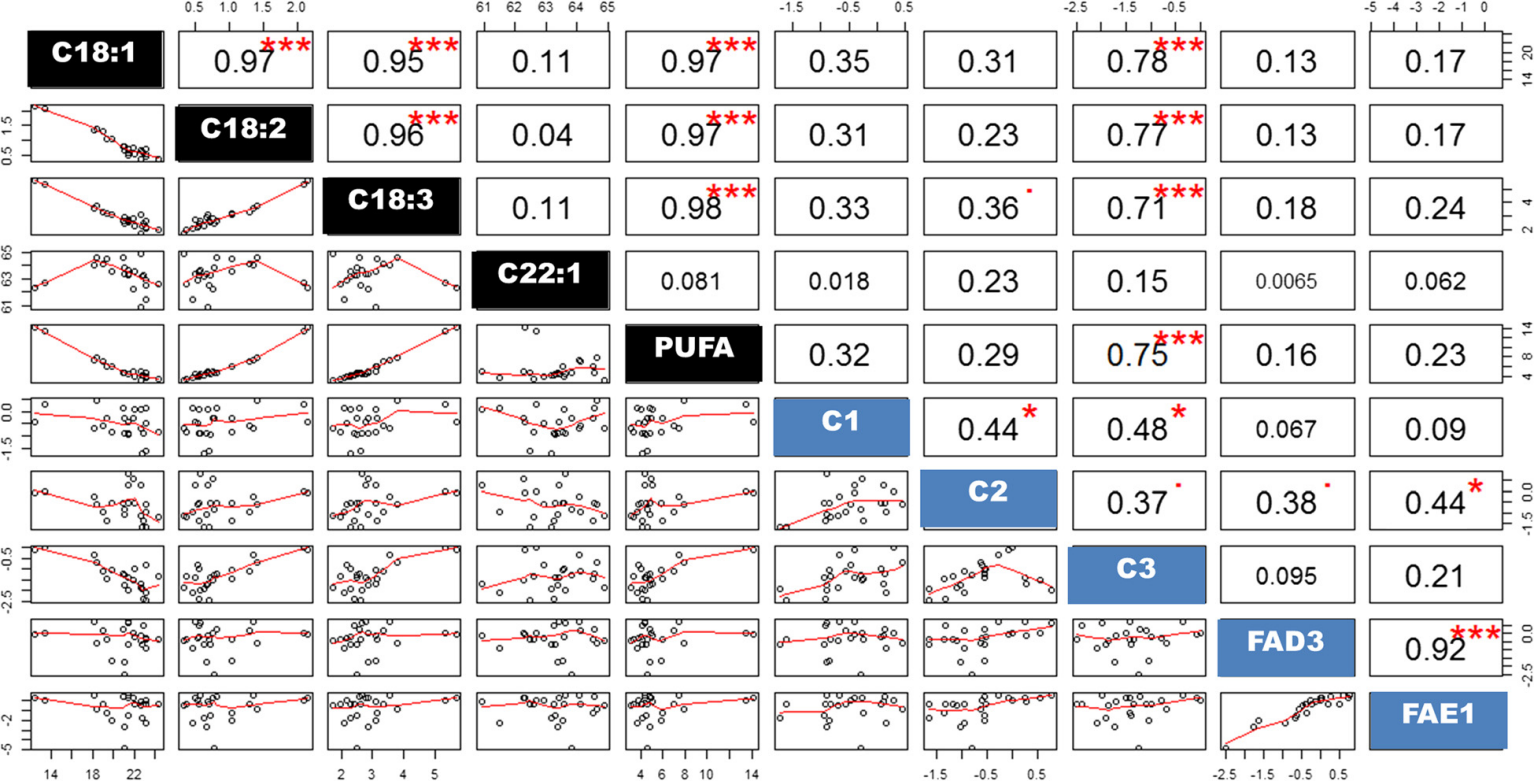
**Correlation plotting between genes expression and oil composition of T**_**2 **_**plants of *C. abyssinica*.** The expression levels and oil compositions were plotted on the lower part; and the values on the upper part represent the absolute values of the correlation coefficient “r”. Significance levels: “^**.**^”, *P* < 0.1; “*”, *P* < 0.05; “**”, *P* < 0.01; “***”, *P* < 0.001.

To investigate the relationship between differential expression levels of the target genes in the T_2_ generation and the oil composition, correlation analysis was carried out. All the correlations and coefficients are plotted in Figure 5. Among the individual T_2_ plants of WG4-4 and WG19-6 that showed differential *CaFAD2* expression levels, some significant correlations were observed for the expression levels of *CaFAD2-C1* to -*C3*, *CaFAD3* and *CaFAE1*. Overall, the expression of *CaFAE1* is co-ordinately regulated with both, *CaFAD3* (r = 0.92, *P* < 0.001) and *CaFAD2-C2* (r = 0.44, *P* < 0.05) (Figure 5). These correlations are much stronger in the WG4-4 family (r = 0.943 and 0.957 respectively). Regarding the oil composition, the contents of both C18:2 and C18:3 were negatively related (r = -0.97 and -0.95 respectively, *P* < 0.001) to the C18:1 content. However, the C22:1 content was not significantly related to the observed changes in other oil compounds (*P* > 0.1) (Figure 5). In accordance with its function in the oil biosynthesis pathway, the expression level of *CaFAD2-C3* is negatively related to the C18:1 content (r = -0.78, *P* < 0.001) and positively related to the contents of C18:2 (r = 0.77, *P* < 0.01) and C18:3 (r = 0.71, *P* < 0.01) (Figure 5). However, there were no correlations found between these fatty acids and the expression of other genes (Figure 5).

## Discussion

*C. abyssinica*, an under-utilized crop, has limited genetic variation and the improvement of crambe oil by traditional breeding has reached a bottleneck. Recently, using a genetic engineering approach, crambe lines with a desirable oil type have been obtained by silencing *CaFAD2* genes [29]. In *C. abyssinica*, three functional *FAD2* genes are present (*CaFAD2-C1* to -*C3*) [32]. Even though the functional defect of one of the family members may be complemented by the other members of the gene family [41], one or few of these family members usually play a predominant role, so that a primary functional analysis of gene family members is normally required prior to mutation breeding [42]. Our study was initiated to characterise the effect of the three functional *CaFAD2* genes (*CaFAD2-C1* to -*C3*) to determine the appropriate targets for a targeted mutation approach (TILLING) to develop non-GM crambe lines with novel oil types.

Here we used a construct containing a part of the *CaFAD2-C2* sequence, which is 96% to 97% identical to the sequences of other expressed *CaFAD2* genes, to obtain seed-specific silencing (*Napin* promoter) of the endogenous *CaFAD2* gene family members in crambe. The results proved that one RNAi trigger is able to affect the expression of multiple members of the gene family but in different patterns (Figure 1). In T_2_-plants, the main *FAD2* gene expressed in crambe seeds, *CaFAD2-C3,* is strongly silenced whereas the two lower expressed genes, *CaFAD2-C2* and *-C1* (respectively 4 and 100 times lower than *CaFAD2-C3* at 20 DAP [32]), are silenced to different levels ranging from strong- to zero silencing. The reason why no reduction of *CaFAD2* gene expression was detected in two T_1_-plants (WG19-5 and WG19-6), which nevertheless showed significant difference for seed-oil composition, is probably due to the heterozygous nature of the seed samples. This can cause that segregating wild-type seeds can be present in the seed bulks used for expression analysis, whereas the oil composition was measured on single seeds. Another possibility is that silencing in these lines took place in the developing seeds at other time points beyond the expression peak at 20 days after pollination (DAP). Indeed, the results of Southern analysis do suggest that the T_1_-plant WG19-6 is heterozygous, because a varying number of transgene insertions was detected in its offspring (T_2_), and also that WG4-4 (T_1_-plant) is most likely homozygous because a single transgene insertion was observed in all randomly tested progeny (T_2_) of WG4-4 (Additional file 2).

Over two generations (T_1_ and T_2_), the expression patterns of *CaFAD2* in WG4-4, carrying a single transgene insertion, were similar whereas differences in silencing levels over generations were observed for WG19-6. The multiple transgene insertions in WG19-6 may cause different types of variation in the T_2_ population, which may influence the level of silencing. It is known that trace amount of dsRNA is sufficient to trigger gene silencing [43] and the degree of silencing has no association with insert copy number [44]. Therefore, a plausible explanation to different silencing levels over T_1_ and T_2_ generations of WG19-6 is that a higher level of homozygosity in the seeds of the T_2_ generation leads to a lower number of segregating wild type seeds, which is of importance if the seeds are analysed for expression “in bulk”.

The T_2_ progenies of two FAD2-RNAi plants (WG4-4 and WG19-6) were tested in detail for the level of gene expression of the different *CaFAD2* gene family members (Figure 2A and B) and for oil composition (Figure 3 and 4). The results showed that the silencing of gene expression is stably inherited to the subsequent generation. The stability of RNAi-silencing was also reflected at the seed-oil composition, for instance, in the T_2_-progeny of WG4-4, the C18:1 level was significantly higher and PUFA level was significantly lower than those of the control plants (Table 1 and Figure 3). The values of important individual oil compounds are close to the average of the parental T_1_-plants for both lines, WG4-4 and WG19-6, indicating that the high C18:1 and low PUFA traits were stably transmitted to the subsequent generation.

### Correlation and expression regulation analysis

Compared to *CaFAD2-C1* and *CaFAD2*-*C2*, *CaFAD2-C3* was strong and stably silenced by the seed specific RNAi trigger. On average, silencing of *CaFAD2-C3* is constantly significant in both T_2_ families (offspring of WG4-4 and WG19-6). Although some T_2_-plants show a clear silencing of *CaFAD2-C1* and *CaFAD2-C2*, the overall family average of these genes are not significantly different from the control for both families. Consequently, the only significant difference in gene expression observed between the two T_2_ families is the difference in *CaFAD2-C2* expression (Figure 2B).

Taken together with the fact that a change in oil composition was found in all T_2_-plants and that out of the three *CaFAD2* genes *CaFAD2-C3* is the highest expressor in developing crambe seeds [32], it is most likely that this gene plays a direct role in seed oil synthesis. This idea is supported by the study on the seed-oil of individual T_2_-plants (Figure 5). Based on the differential silencing of *CaFAD2-C2* among T_2_ plants of both families and the silencing of *CaFAD2-C1* observed in several individual T_2_ plants, there is no clear evidence showing that the expression of these two genes is correlated to changes in the seed oil composition (Figure 5). Furthermore, the changes in oil composition due to *CaFAD2-C3*-silencing were not complemented in individual T_2_-plants in which *CaFAD2-C1* and *CaFAD2-C2* were unaffected and expressed to wild-type levels.

The trait of high C18:1 is controlled by quantitative trait loci and thus needs coordinated regulation of multiple loci [21]. Previous studies proved that the *FAD2* gene plays an essential role for this trait [9,38]. In addition, *FAD3*, *FAE1* and other loci with minor effect are likely required to establish high C18:1 pool during oil biosynthesis [21,45]. In this study, we also investigated the regulation of *FAD3* and *FAE1* while silencing the *CaFAD2* genes. It is known that *FAD3* and *FAE1* are regulated by abscisic acid [46]. Herein, we found that the expression of *FAE1* is positively related to that of *FAD3* and one of the *FAD2* genes, *CaFAD2-C2* (Figure 5). This finding is consistent to previous studies in which these functionally related genes (*FAD3* and *FAE1*) showed coordinated regulation in Arabidopsis and *Brassica napus*[46,47]. However, it is unknown why the other two *CaFAD2* genes do not show a similar co-regulation with *FAD3* and *FAE1*.

### The *CaFAD2-C3* gene is a target gene for crambe oil improvement by mutation breeding

Here, we show that *CaFAD2-C3* is the main *CaFAD2*-gene involved in determining the C18:1 and PUFA contents in crambe oil (Figure 5). Embryo tissue is the main compartment of oil storage in crambe seed, so the oil composition in the seeds is mainly determined by that in the embryo. Therefore, *CaFAD2-C3* is most likely responsible for fatty acid synthesis in the embryo. The other two genes, *CaFAD2-C1* and *CaFAD2-C2*, might be responsible to change fatty acid contents in other seed compartments (e.g. seed coat and endosperm). For instance in olive (*Olea europaea*), two FAD2 genes (*OeFAD2-1* and *OeFAD2-2*) are expressed both in two seed compartments, seed coat and embryo. Of these two *OeFAD2* genes, the expression of *OeFAD2-2* was positively correlated to C18:2 content in the seed coat rather than in the embryo under cold condition [48].

## Conclusions

The finding that the prominent role of *CaFAD2-C3* is further substantiated by the observation that the effect on oil-composition caused by *CaFAD2-C3* silencing is not restored in individual T_2_-plants with wild-type expression of *CaFAD2-C2* and -*C1* respectively. This finding suggests that *CaFAD2-C3* may provide an important target gene for TILLING and mutation breeding aiming at pronounced changes of C18:1 and PUFA in crambe oil.

## Methods

### Plant materials

*C. abyssinica* cv. 'Galactica’ was previously transformed with an RNAi construct (pWatergate) [29]. This construct contains an inverted repeat (IR) of the *CaFAD2-C2* coding sequence (355 base pairs for each IR part, 97% identical to *CaFAD2-C1* and 96% identical to *CaFAD2-C3*) following a seed specific promoter (*Napin*) (Additional file 4). The plant used as control was transformed with an empty construct (pRCNG) which contains no genes involved in fatty acids biosynthesis [49]. Two T_0_ lines were used to develop six T_1_ plants/lines (WG4-3, WG4-4, WG4-5, WG19-4, WG19-5 and WG19-6), grown in the greenhouse with temperature of 22°C and photoperiod of 16 h. From these T_1_ plants, the developing seeds at 20 DAP (days after pollination) were collected for RNA isolation for gene expression analysis and ripe seeds were harvested for oil composition analysis. Seeds of two T_1_-lines, WG4-4 and WG19-6, were used to produce T_2_-plants. The young leaves of T_2_-plants were collected for Southern analysis. The developing seeds (20 DAP) and ripe seeds of the T_2_-plants were harvested for RNA isolation and oil composition analysis respectively.

### Quantitative reverse transcription PCR (qRT-PCR) and data analysis

To examine the level of gene-silencing, the expression of the individual crambe FAD2 genes, *CaFAD2-C1*, *CaFAD2-C2* and *CaFAD2-C3*, was measured in six T_1_ plants (WG4-3, WG4-4, WG4-5, WG19-4, WG19-5 and WG19-6) on bulks of five to ten developing seeds (20 DAP, days after pollination) and compared to their expression level in the control (plant transformed with an empty construct). The primers specific for the different crambe *FAD2* genes were developed based on their sequences in *C. abyssinica* cv. 'Galactica’ (GenBank: JX964743, JX964744, JX964745), and the primers of *FAD3* and *FAE1* were developed on the sequences of *AtFAD3* (GenBank: 42570333) and crambe *FAE1* (GenBank: 60543786). The gene *β-actin* 2 (GenBank: 20465834) was used as a reference gene. All primers used in qRT-PCR are listed in Additional file 5. Total RNA was extracted from bulked seeds of T_1_-and T_2_ -plants respectively (5 to 10 seeds per T_1_-plant and per T_2_-plant, 20 DAP) with RNeasy Plant Mini Kits (Qiagen, Germany) according to the manufacturer’s instructions. The isolated RNA was treated with RNase-free TURBO DNase (Ambion, USA) to remove residual genomic DNA. First-strand cDNA was synthesized in 20 μl from 1 μg of total RNA with iScript™ cDNA Synthesis Kit (Bio-rad, USA), in parallel 1 μg of RNA of each sample was treated in the same way but without adding reverse transcriptase, as negative controls (RT-). The cDNA was 20 × diluted and used as templates for real-time PCR. The PCR reaction contains 2 μl templates, 5 μl SYBR Green Super Mix (Bio-rad, USA), and 1 μl of each of the forward and reverse primers (3 μM) in total 10 μl reaction. Cycling conditions were 1 cycle at 95°C for 3 min followed by 30 cycles at 95°C for 10 s, 60°C for 1 min, then a final melt step from 65°C to 95°C ramp with 0.5°C increments per cycle to monitor specificity. PCR reactions were performed in triplicate. The expression of each replicate was normalized by the reference gene, *β-actin* 2, which has shown to be stably expressed in both, crambe seedlings under arsenate stress and various *Brassica napus* cultivars [47,50]. The relative expression level of each replicate was calculated according to the comparative CT method (User bulletin no. 2, ABI PRISM 7700 Sequence Detection System, December 1997; Perkin-Elmer, Applied Biosystems). The mean of three replicates represents the relative expression level of a line.

### Oil composition and correlation analysis

The fatty acid (FA) fraction was extracted from single crambe seeds, and fatty acid methyl esters (FAMEs) were analysed using gas chromatograph (GC, column/DB-23, Agilent). For each T_1_-plant 20, and for each T_2_-plant 10 individual ripe seeds were collected respectively per plant for fatty acid extraction. The pods of single seeds were removed and then crushed in a plastic tube with screw cap and 300 μl of hexane and 40 μl of KOH (5 M)/methanol were added, followed by vortexing and heating samples at 60°C for 6 min. The samples were then allowed to cool down to room temperature before centrifugation at 3000 rpm for 5 min, the upper layer was used for GC. The extraction (1 μl) was injected into GC with split ratio 1:20 and the condition of oven was 180°C for 10 min, ramp to 240°C for 7 min with 7.5°C increments per min. The mean percentages of FA compounds of each line were calculated from the average of 20 or 10 single seed values. The data of gene expression and oil composition in the seeds of T_2_ generation were used for correlation analysis. These data were plotted and a Pearson correlation was run to determine correlation with R package [51].

### Southern analysis

Genomic DNA was isolated from young leaves of T_2_ plants with the method described by Aldrich and Cullis [52] but with 1% (w/v) polyvinylpyrrolidone-10 in the DNA extraction buffer. A probe (686 base pairs) was designed on the *nptII* gene in the WG construct and labelled with [^32^P]ATP. The primers for nptII probe preparation are listed in Additional file 5.

For copy number determination, a total of 40 μg of DNA extracted was digested overnight with *Dra* I, an enzyme that cuts the T-DNA in a site outside the probe sequence, fractionated on 0.8% agarose gel and transferred to Hybond N + membrane (Amersham Biosciences, UK) according to the manufacturer’s recommendations. The procedures of hybridization and visualization were performed as described by Cheng et al. [32].

## Competing interests

The authors declare that they have no competing interests.

## Authors’ contributions

JC, EMJS and ENVL designed the experiments. JC carried out the expression analysis and wrote the draft manuscript; EMJS performed Southern analysis and amended the manuscript. LHZ and JG provided transgenic plants and commented the manuscript. ACD and WQ performed GC analysis. BH, FAK and RGFV convinced the study and participated in amending the draft manuscript. All authors read and approved the final manuscript.

## Supplementary Material

Additional file 1 **The levels of C18:1 (A) and PUFA (B) in the single seeds of T**_**1**_** plants.** For each plant, around 20 single seeds were measured. One point represents a single seed. Click here for file

Additional file 2 **Determination of transgene copy number for T**_**2**_** plants of *C. abyssinica* by Southern analysis.** The blotting membrane was hybridized with ^[32P]^ATP-labelled nptII probe. The family WG19-6 harbours 4 or 5 transgene insertions, and the family WG4-4 harbours a single insertion. Lane 1-12: WG19-6-17; WG19-6-14; WG19-6-13; WG19-6-11; WG19-6-10; WG19-6-9; WG19-6-8; WG19-6-7; WG19-6-5; WG19-6-4; WG19-6-2; WG19-6-1. Lane 13–17: WG4-4-13; WG4-4-10; WG4-4-5; WG4-4-3; WG4-4-1. Lane C1-C2: two wild-type controls. Click here for file

Additional file 3 **The plot of oil composition (C18:1 vs. PUFA) in the seeds of T**_**2**_** plants of family WG4-4, WG19-6 and the control (CTRL).** Each point represents a single seed of T_2_-plant. Click here for file

Additional file 4 **Schematic diagram of T-DNA region of the RNAi construct (not to scale).** LB and RB, T-DNA left border and right border, respectively. Napin Promoter, seed specific promoter from *Brassica napus*. The attB1 and attB2, recombination sites used in BP reaction of Gateway^®^. CaFAD2-IF and CaFAD2-IR, 355 base pairs inverted repeats of crambe FAD2-C2 sequence in forward and reverse orientations, the sequence identities to the genes *CaFAD2-C1*,-*C2* and-*C3* are 96%, 99% and 96% respectively. *nptII*, neomycin hosphotransferase II gene. The broken line represents the sequence that forms stem in hairpin RNA. The arrow indicated npt II probe (686 base pairs) for hybridization in this study. Click here for file

Additional file 5 The primers used in this study. Click here for file
