# Supplementary figures and images for: Functional analysis of the omega-6 fatty acid desaturase (CaFAD2) gene family of the oil seed crop Crambe abyssinica
Source: BMC Plant Biol. 2013 Oct 1;13:146. doi: 10.1186/1471-2229-13-146 (PMC3829706; doi:10.1186/1471-2229-13-146)

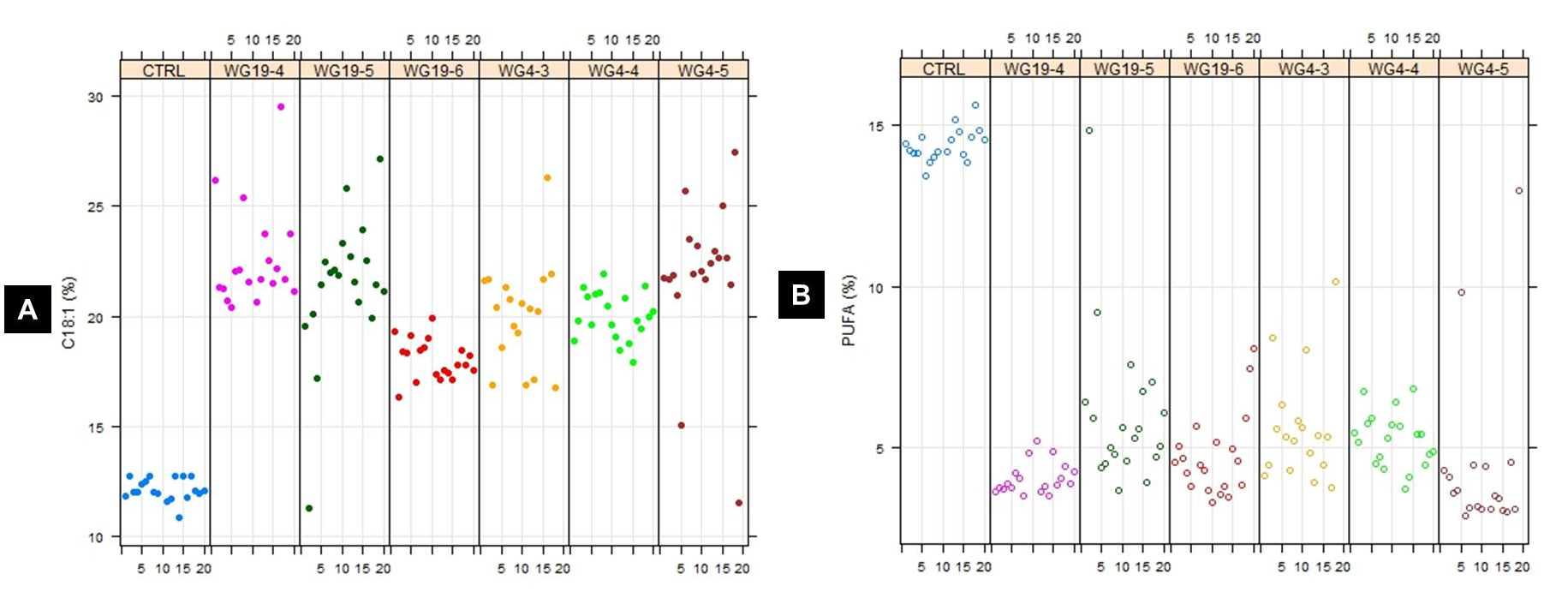

Supplement: Additional file 1 — The levels of C18:1 (A) and PUFA (B) in the single seeds of T 1 plants. For each plant, around 20 single seeds were measured. One point represents a single seed. [file 1471-2229-13-146-S1.png]

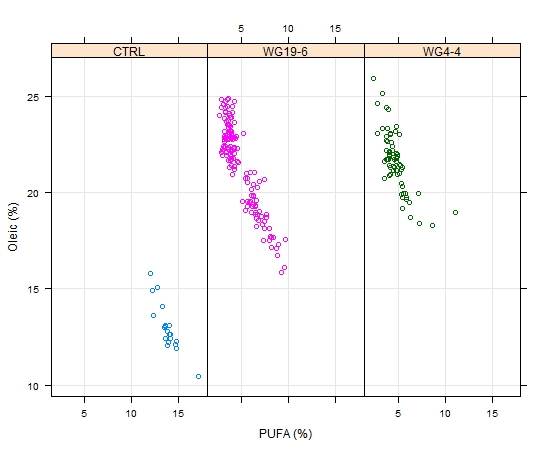

Supplement: Additional file 2 — Determination of transgene copy number for T 2 plants of C. abyssinica by Southern analysis. The blotting membrane was hybridized with [32P]ATP-labelled nptII probe. The family WG19-6 harbours 4 or 5 transgene insertions, and the family WG4-4 harbours a single insertion. Lane 1-12: WG19-6-17; WG19-6-14; WG19-6-13; WG19-6-11; WG19-6-10; WG19-6-9; WG19-6-8; WG19-6-7; WG19-6-5; WG19-6-4; WG19-6-2; WG19-6-1. Lane 13–17: WG4-4-13; WG4-4-10; WG4-4-5; WG4-4-3; WG4-4-1. Lane C1-C2: two wild-type controls. [file 1471-2229-13-146-S2.jpeg]

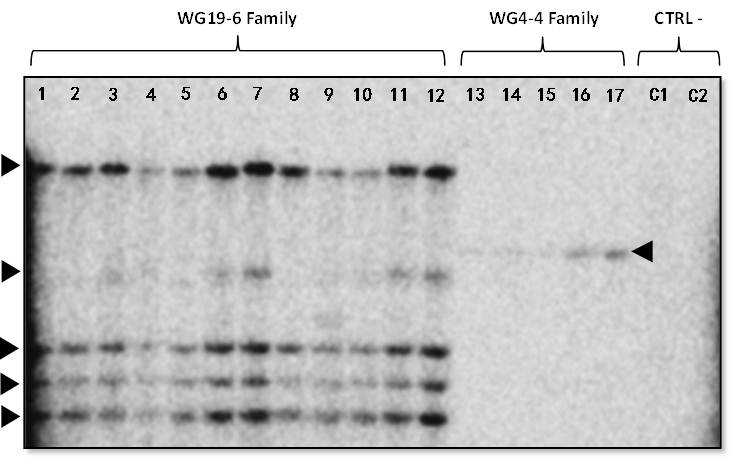

Supplement: Additional file 3 — The plot of oil composition (C18:1 vs. PUFA) in the seeds of T 2 plants of family WG4-4, WG19-6 and the control (CTRL). Each point represents a single seed of T2-plant. [file 1471-2229-13-146-S3.jpeg]

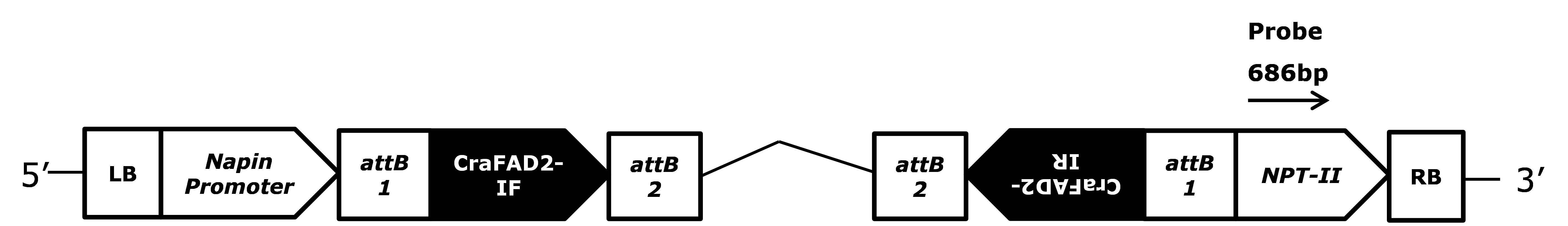

Supplement: Additional file 4 — Schematic diagram of T-DNA region of the RNAi construct (not to scale). LB and RB, T-DNA left border and right border, respectively. Napin Promoter, seed specific promoter from Brassica napus. The attB1 and attB2, recombination sites used in BP reaction of Gateway®. CaFAD2-IF and CaFAD2-IR, 355 base pairs inverted repeats of crambe FAD2-C2 sequence in forward and reverse orientations, the sequence identities to the genes CaFAD2-C1,-C2 and-C3 are 96%, 99% and 96% respectively. nptII, neomycin hosphotransferase II gene. The broken line represents the sequence that forms stem in hairpin RNA. The arrow indicated npt II probe (686 base pairs) for hybridization in this study. [file 1471-2229-13-146-S4.png]
