# Supplementary material for: Functional analysis of the omega-6 fatty acid desaturase (CaFAD2) gene family of the oil seed crop Crambe abyssinica
Source: BMC Plant Biol. 2013 Oct 1;13:146. doi: 10.1186/1471-2229-13-146 (PMC3829706; doi:10.1186/1471-2229-13-146)
Supplement: Additional file 5 — The primers used in this study. [file 1471-2229-13-146-S5.docx]

**Additional file 2. The primers used in this study.**

| Category | Gene name | Forward primer | Reverse primer | Product size (bp) |
| --- | --- | --- | --- | --- |
| Real-time PCR | *CraFAD2-C1* | CAAGGCTGTGTCCTAAGA | CGTCGTCTCTTTCAAGCGGA | 200 |
|  | *CraFAD2-C2* | CGCCATTCCAACACTGGCTCC | GCGAAGCCGTCGTAAGGTCT | 191 |
|  | *CraFAD2-C3* | CAAGGCTGTGTCCTAAGC | AACACTTCATCTCTTTCCAAT | 200 |
|  | *FAD3* | TTGTGATGTGGTTGGATGCT | TGAGGGATTTGTGGGAAGAG | 196 |
|  | *FAE1* | GTGCTGGCGTTATAGCCATT | AGGCTTGTTGGAGAGCAAAA | 191 |
|  | *β-actin 2* | ATTCAGATGCCCAGAAGT | CTCATACGGTCAGCGATA | 180 |
|  |  |  |  |  |
| Probe | *npt-II* | TGGGCACAACAGACAATCGGCTGC | TGCGAATCGGGAGCGGCGATACCG | 686 |
